# Adsorption Performance of Cu-Fe Bimetallic-Modified Coconut Shell Activated Carbon for Ultra-Low-Concentration SO_2_

**DOI:** 10.3390/ma19132811

**Published:** 2026-07-02

**Authors:** Mingjing Zhu, Xiaohui Chen

**Affiliations:** 1 School of Chemical Engineering, Fuzhou University, Fuzhou 350116, China; 18355572743@163.com; 2 National Engineering Research Center for Fertilizer Catalysts, Fuzhou University, Fuzhou 350108, China

**Keywords:** sulfur dioxide, coconut shell activated carbon, Cu-Fe bimetallic, adsorption, deactivation analysis

## Abstract

In this study, Cu-Fe bimetallic-supported adsorbents were prepared using alkali-activated coconut shell activated carbon (AC-OH) as a carrier by impregnation method. To optimize the adsorption effect, the effects of the second metal type, Fe loading amount, Cu/Fe ratio, and operating conditions on the adsorption effect of extremely low-concentration SO_2_ (1 ppm) were systematically investigated. The results showed that when the Cu loading was 5% by mass and the Fe loading was 3% by mass, the adsorbent exhibited optimal adsorption performance, with a breakthrough time of up to 36.5 h and a corresponding breakthrough sulfur capacity of 14.424 mg/g. Further exploration of the conditions shows that the coexistence of O_2_ and H_2_O can significantly promote the adsorption of SO_2_, while reducing the space velocity is beneficial for prolonging the breakthrough time. In terms of regeneration stability, after two adsorption–regeneration cycles, the adsorption activity of the adsorbent decreased to 72.7% of the fresh sample, and the deactivation was mainly attributed to the accumulation of sulfate species and the loss or aggregation of active components. By combining XRD, FT-IR, XPS, SEM and other characterization techniques, the structure–activity relationship and deactivation mechanism of the adsorbent were analyzed. This bimetallic-modified activated carbon has shown great potential for deep purification of extremely low concentrations of SO_2_.

## 1. Introduction

Sulfur dioxide (SO_2_) is a common atmospheric pollutant that poses serious threats to human health and the ecological environment. It mainly originates from fossil fuel combustion, municipal solid waste incineration, and industrial production [1,2]. With the effective global control of high-concentration SO_2_ emission sources, the concentration of sulfur-containing substances in the environment has decreased significantly. However, the potential environmental and health risks of ultra-low-concentration SO_2_ in the air (typically referring to concentrations below 100 ppm, especially less than 10 ppm) remain non-negligible [3]. In recent years, significant progress has been made in high-concentration SO_2_ removal technologies, primarily including wet flue gas desulfurization processes such as the limestone–gypsum method, ammonia method, and magnesium method [4,5], as well as semi-dry techniques like circulating fluidized bed and spray drying. These technologies demonstrate advantages of high removal efficiency and stable operation in the treatment of typical high-concentration flue gas [6,7,8]. However, when applied to the deep removal of extremely low-concentration SO_2_, these processes generally expose problems such as insufficient mass transfer kinetics and low adsorbent utilization efficiency [9,10].

Activated carbon, as a porous adsorbent material, features high specific surface area, well-developed pore structure, and low cost, and has attracted widespread attention in the field of SO_2_ adsorption [11,12]. Nevertheless, the adsorption of SO_2_ on unmodified activated carbon mainly relies on physical interactions with weak affinity, making it difficult to meet the requirements of deep purification [13]. To address this, researchers have systematically investigated the effects of functional groups, chemical composition, and relative humidity on the SO_2_ removal performance of activated carbon. KOH activation can introduce basic oxygen-containing functional groups and enrich the microporous structure, thereby enhancing the adsorption capacity for acidic gases [14,15]. In recent years, it has been found that loading metals can introduce chemisorption sites onto the activated carbon surface. These metal components can convert SO_2_ into sulfates and immobilize them on the material surface through catalytic oxidation, thus significantly enhancing the adsorption capacity [16,17]. However, single-metal-loaded adsorbents still exhibit deficiencies in the number of active sites, dispersion, and resistance to deactivation [18]. Recently, systematic studies have been conducted on activated carbon adsorbents loaded with different bimetallic oxides, and bimetallic systems have received extensive attention owing to the synergistic effects between the components [19,20,21]. Studies have shown that introducing a second metal can modulate the electronic structure of active centers, increase the oxygen vacancy concentration, and improve the dispersion state of metal particles, thereby enhancing desulfurization performance and regeneration stability. Liu et al. [19] found that the Cu-Fe bimetallic loading system significantly reduced the initial oxidation reaction energy barrier of SO_2_ through electron transfer, especially electron transfer with hydroxyl groups, which almost completely eliminated the reaction energy barrier. The effective combination of oxygen-containing functional groups and bimetallic species constructed a channel that promoted the connection between SO_2_ and the material surface, markedly altering the physicochemical environment and enhancing the desulfurization capacity of the material. Xiao et al. [20] prepared Ti/Co bimetallic-loaded adsorbents whose performance far exceeded that of single-metal counterparts, demonstrating excellent regeneration stability. Wang et al. [21] pointed out that bimetallic systems can form superior catalytic active centers (such as Me_1_-O-Me_2_ structures) through geometric and ligand effects, which can compensate for the shortcomings of single metals in complex reactions. Nonetheless, systematic research on bimetallic-modified activated carbon systems for the efficient adsorption of ultra-low-concentration SO_2_ (1 ppm) is still lacking, and the influence mechanisms of the second metal type, metal loading, bimetallic ratio, and reaction conditions (space velocity, atmosphere, etc.) on the adsorption behavior remain unclear [9,13].

Based on this, in this study, KOH-activated coconut shell activated carbon (AC-OH) was used as the support, and bimetallic-loaded adsorbents were prepared via a one-step impregnation method. The effects of the second metal type, the loading amount of the second metal, and the mass ratio of the two metals on the adsorption performance were systematically investigated, and the influences of volumetric space velocity and different atmosphere conditions were explored. Meanwhile, through multiple characterization techniques, the phase structure, surface chemical state, pore structure characteristics, and thermal stability of the adsorbents were thoroughly analyzed to reveal the bimetallic synergistic mechanism. Furthermore, adsorption–regeneration cycle experiments were carried out, and the causes of adsorbent deactivation were discussed in combination with the characterization results before and after the reaction. This work provides a theoretical basis and experimental support for the development of efficient and stable adsorbents for the deep purification of ultra-low-concentration SO_2_.

## 2. Materials and Methods

### 2.1. Adsorbent Preparation

Coconut shell activated carbon (30–60 mesh) was used as the support. It was treated with a KOH solution (4.0 g KOH dissolved in 40 mL deionized water) under heating and stirring at 60 °C for 3 h. After the treatment, the mixture was filtered and washed with deionized water until neutral, and then dried in a vacuum drying oven at 80 °C overnight to obtain the alkali-activated support (denoted as AC-OH). The bimetallic adsorbent was prepared by a one-step impregnation method [19,22]. Appropriate amounts of metal nitrates were sequentially dissolved in 10 mL deionized water, and 0.5 g of AC-OH was added. The mixture was ultrasonically dispersed at room temperature for 30 min, followed by evaporation to dryness in a water bath at 60 °C. The resulting solid was dried in a vacuum oven at 80 °C overnight, and then transferred to a tube furnace and calcined at 300 °C for 2 h under a N_2_ atmosphere to obtain the final product.

### 2.2. Adsorption Performance Evaluation

**Figure 1:**
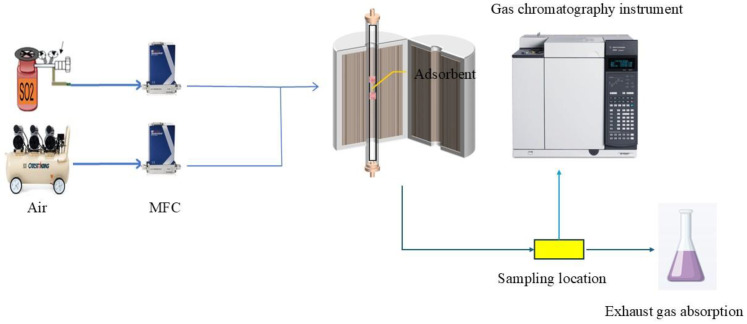
Flow chart of adsorption activity test.

Adsorption experiments were carried out in a fixed-bed reactor at room temperature. The inlet gas contained 1 ppm SO_2_, and the gas hourly space velocity (GHSV) was 60,000 h^−1^ with an adsorbent mass of 0.04 g. Breakthrough was defined when the outlet SO_2_ concentration reached 0.1 ppm. The breakthrough time and sulfur capacity were then calculated. The outlet gas was quantitatively analyzed using a GC-6890N gas chromatograph (California, USA) by the external standard method. An HP-5 capillary column was employed, with a hydrogen flame ionization detector (FID) (California, USA) as the front detector and a PFPD 5380 sulfur detector (California, USA) as the rear detector. Nitrogen was used as the carrier gas, and the injection volume was 1 mL. The adsorption flow diagram is shown in Figure 1.

### 2.3. Adsorbent Characterization

The phase structure was analyzed using an X-ray powder diffractometer (PANalytical, Almelo, The Netherlands, X’pert Pro MPD with Cu Kα radiation). The surface functional groups were characterized by Fourier transform infrared spectroscopy (Thermo Fisher Scientific, Waltham, MA, USA, Nicolet 6700). The elemental valence states were examined by X-ray photoelectron spectroscopy (Waltham, MA, USA, EscaLab 250Xi). The morphology was observed using a field-emission scanning electron microscope (ZEISS, Oberkochen, Germany, GeminiSEM 300). The elemental composition of the sample microareas was determined by energy-dispersive X-ray spectroscopy (Oxford Instruments, Oxford, UK, Xplore 30). The specific surface area and pore structure were measured with an N_2_ adsorption–desorption analyzer (Micromeritics, Norcross, GA, USA, ASAP 2020M). The thermal stability and compositional changes of the samples were investigated by thermogravimetric analysis (Netzsch, Selb, Germany, STA 449).

## 3. Results and Discussion

### 3.1. Effect of the Second Metal Type on Adsorption Performance

**Figure 2:**
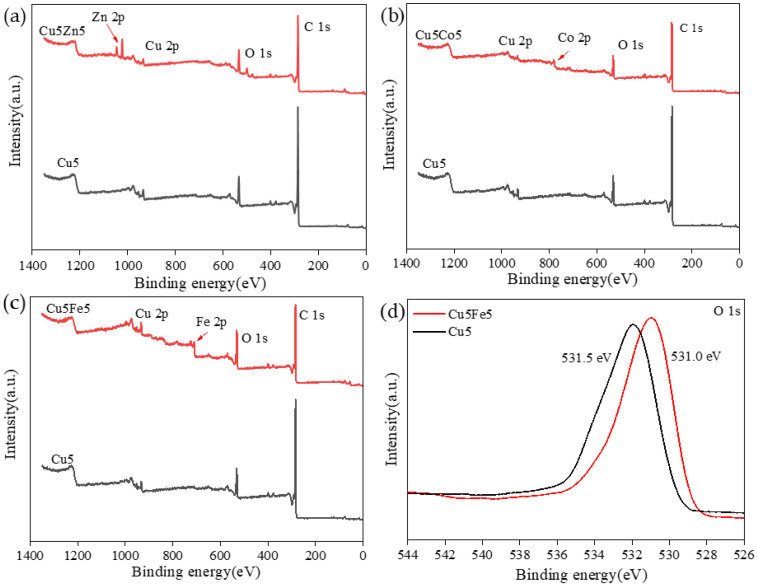
XPS spectra of modified activated carbon with different metal loadings. (**a**) Cu5Zn5, (**b**) Cu5Co5, (**c**) Cu5Fe5, (**d**) Comparison of oxygen peaks between Cu5Fe5 and Cu5 adsorbents.

Figure 2 presents the survey XPS full spectra of different bimetallic adsorbents (Cu5Zn5, Cu5Co5, Cu5Fe5) and the comparison of O 1s spectra between Cu5Fe5 and Cu5. Figure 2a shows the survey XPS spectrum of Cu5Zn5, in which the characteristic peaks of Zn 2p, Cu 2p, O 1s, and C 1s can be identified. In Figure 2b, besides the peaks of Cu 2p, O 1s, and C 1s, the characteristic peak of Co 2p is clearly observed, indicating its successful loading onto the adsorbent. Figure 2c displays the survey XPS spectrum of Cu5Fe5, where the characteristic peaks of Cu 2p, Fe 2p, O 1s, and C 1s are observed. The appearance of the Fe 2p peak confirms the successful incorporation of iron onto the material. Based on the shape and position of the Cu 2p peak, it can be inferred that a strong electronic interaction exists between iron and copper species, possibly forming bimetallic active centers that are beneficial for enhancing the adsorption performance. Figure 2d compares the high-resolution O 1s spectra of Cu5Fe5 and Cu5. The O 1s peak for the Cu5 sample is located at approximately 531.5 eV, whereas that for Cu5Fe5 appears at 531.0 eV. The decrease in binding energy indicates that the introduction of iron alters the chemical environment of the oxygen species. This phenomenon may be associated with the formation of Fe-O-Cu structures that induce electron redistribution, or with the generation of more oxygen vacancies, resulting in oxygen species in a more electron-rich chemical state, thereby facilitating the adsorption reaction [23,24].

**Figure 3:**
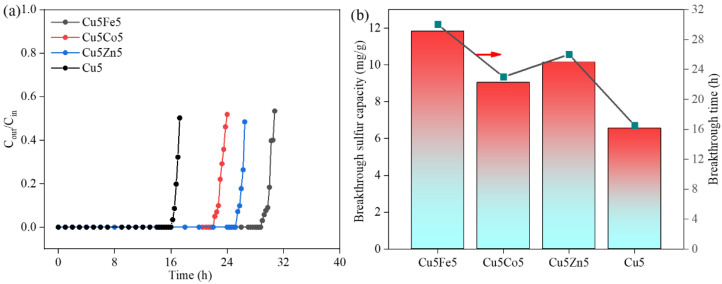
The adsorption effect of different bimetallic-loaded adsorbents. (**a**) Breakthrough curve; (**b**) breakthrough time and breakthrough sulfur capacity. Among them, the bar chart corresponds to the breakthrough sulfur capacity, and the line chart corresponds to the breakthrough time.

Based on the original monometallic Cu-based adsorbent, a series of bimetallic adsorbents were prepared by introducing different second-metal components. Figure 3 presents the adsorption performance of these bimetallic adsorbents, where Figure 3a shows the breakthrough curves and Figure 3b displays the breakthrough time and breakthrough sulfur capacity. As shown in Figure 3a, the adsorption performance follows the order Cu5Fe5 > Cu5Zn5 > Cu5Co5 > Cu5. The breakthrough sulfur capacity and breakthrough time of the Cu5Fe5 adsorbent were 11.832 mg/g and 30 h, respectively; for the Cu5Zn5 adsorbent they were 10.146 mg/g and 26 h; and for the Cu5Co5 adsorbent they were 9.047 mg/g and 23 h. Among them, Cu5Fe5 adsorbent exhibited the best adsorption activity. Compared with the monometallic adsorbent, the bimetallic adsorbents exhibited a certain synergistic effect. The introduction of Fe enhanced the dispersion of the active components and increased the number of effective adsorption sites, thereby strengthening the adsorption capacity of the adsorbent for ultra-low-concentration SO_2_ [25,26].

### 3.2. Effect of Second-Metal Loading on Adsorption Performance

**Figure 4:**
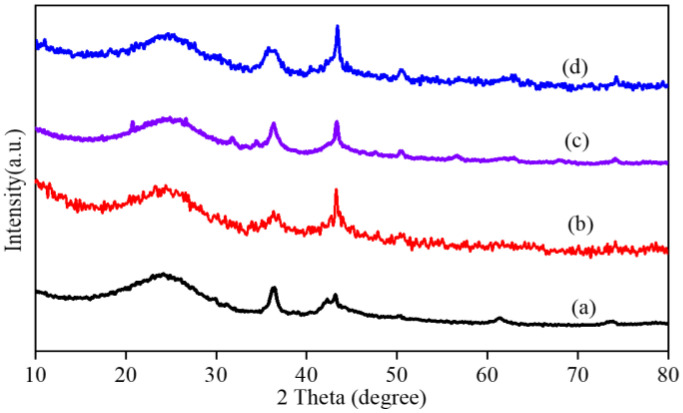
XRD spectra of Cu5Fex bimetallic adsorbent prepared with different iron loadings. (a) Cu5, (b) Cu5Fe1, (c) Cu5Fe3, (d) Cu5Fe5.

Figure 4 presents the XRD patterns of Cu5 and the adsorbents prepared with different iron loadings. It can be observed that all samples (Cu5, Cu5Fe1, Cu5Fe3, and Cu5Fe5) exhibit diffraction peaks similar to those of activated carbon materials. The peaks located at approximately 23° and 43° correspond to the (002) and (100) crystal plane diffractions of activated carbon [27], and their peak positions show no significant shift. As shown in pattern (a), the peaks at 35.4°, 53.4°, 61.5°, and 75.2° correspond to the characteristic diffraction peaks of the (002), (020), (−113), and (311) planes of copper oxide, respectively. From patterns (b) to (d), it can be seen that with increasing iron loading, the diffraction peak at 35.4° broadens further, and its position corresponds to the (110) crystal plane diffraction of α-Fe_2_O_3_ at 35.6°. Meanwhile, the characteristic diffraction peak signal of the (100) crystal plane at 43° is significantly enhanced and broadened simultaneously, indicating a decrease in the crystallite size of the samples. This change is mainly attributed to the introduction of iron oxides, which promotes the local graphitization of the originally disordered carbon crystallites [28].

**Figure 5:**
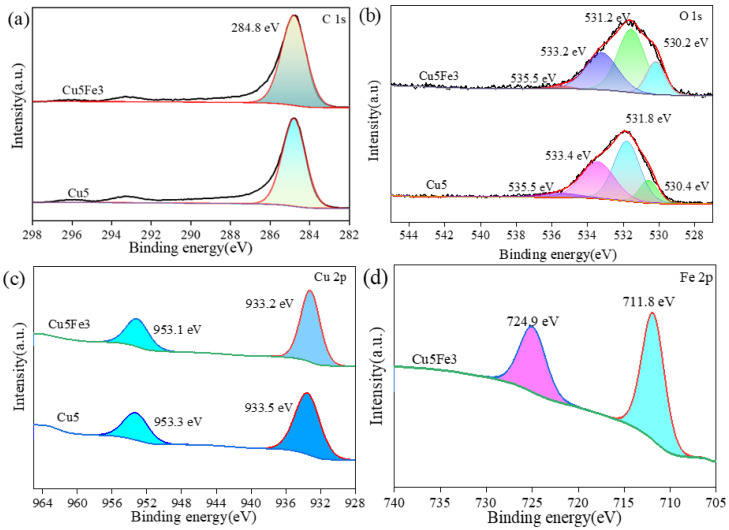
XPS fine spectra of C, O, Cu, and Fe elements for Cu5 and Cu5Fe3 adsorbents. (**a**) C 1s, (**b**) O 1s, (**c**) Cu 2p, (**d**) Fe 2p.

To further investigate the effect of iron loading on the chemical states of the surface elements, high-resolution XPS spectra of C, O, Cu, and Fe for the Cu5 and Cu5Fe3 adsorbents were analyzed, and the results are presented in Figure 5.

Figure 5a shows the high-resolution C 1s spectra of the two materials. Both adsorbents exhibit a prominent characteristic peak at 284.8 eV, which is assigned to C-C and C-H species in the carbon framework. The binding energy positions of the two adsorbents show no significant shift, indicating that the carbon skeleton structure is not substantially disrupted.

Figure 5b presents the high-resolution O 1s spectra. According to the peak fitting results, the binding energy has shifted from 530.4 eV to 530.2 eV, and the peak area attributed to metal lattice oxygen has significantly increased. This change is primarily due to the alteration of the chemical environment of the oxygen species on the adsorbent surface after Fe loading, leading to the formation of lattice oxygen in metal oxides (Fe-O-Cu) at a lower binding energy.

Figure 5c displays the high-resolution Cu 2p spectra of Cu5 and Cu5Fe3. Both samples exhibit the spin–orbit splitting doublets of the Cu 2p orbital, corresponding to the Cu 2p_3/2_ and Cu 2p_1/2_ main peaks, respectively. After Fe loading, the Cu 2p_3/2_ peak shifts from 933.5 eV to 933.2 eV. This negative shift indicates a slight increase in the electron cloud density around copper after iron loading, possibly due to electronic interactions and charge transfer between Fe and Cu, which alters the chemical environment of copper.

Figure 5d shows the high-resolution Fe 2p spectra of the two samples. The Cu5Fe3 sample displays distinct Fe 2p characteristic peaks, confirming the successful loading of iron. The main characteristic peak is located at 711.8 eV and is assigned to the Fe 2p_3/2_ orbital. Meanwhile, the Fe 2p_1/2_ peak is observed at approximately 725 eV, and the energy separation between this peak and the main peak is consistent with the characteristics of ferric compounds such as α-Fe_2_O_3_ [29,30].

**Figure 6:**
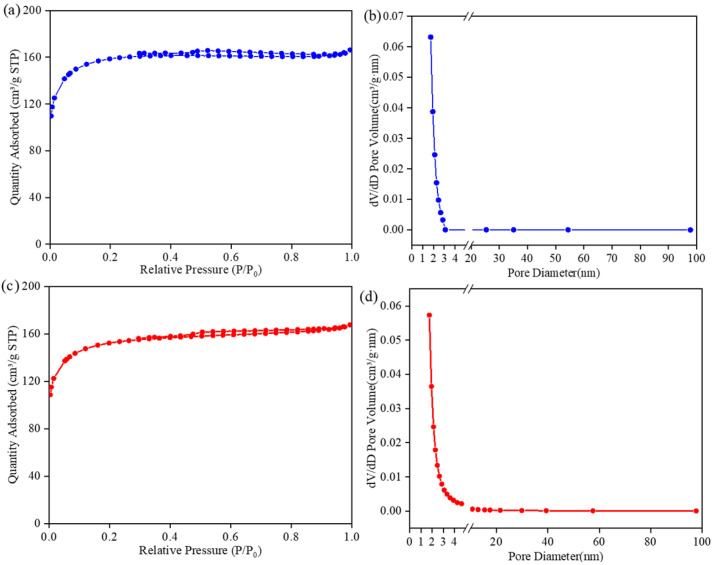
The nitrogen adsorption desorption isotherm curve and pore size distribution of Cu5 and Cu5Fe3 adsorbent. (**a**) Cu5 adsorption isotherms, (**b**) Cu5 pore size distribution curves, (**c**) Cu5Fe3 adsorption isotherms,(**d**) Cu5Fe3 pore size distribution curves.

Figure 6 presents the N_2_ adsorption–desorption isotherm and the corresponding pore size distribution of the Cu5Fe3 and Cu5 adsorbent. As can be seen from the figure, the isotherm exhibits typical Type I characteristics accompanied by an H4 hysteresis loop in the relative pressure range of 0.4–1.0, indicating a microporous–mesoporous structure. The pore size distribution reveals that the pores of the loaded material are predominantly micropores, with a small quantity of mesopores also present [31,32,33].

Table 1 lists the specific surface area (S_BET_), pore volume (V_BJH_), and average pore diameter (d_BJH_) of the Cu5 and Cu5Fe3 adsorbents. It can be observed that after further increasing the iron loading, both the specific surface area and the pore volume of the adsorbent decreased to varying degrees, indicating that the second metal was successfully loaded onto the adsorbent and further occupied the pore channels of the activated carbon. Notably, the specific surface area decreased only slightly, suggesting that the iron species are uniformly dispersed on the Cu5 support without significant agglomeration or pore blockage. The average pore diameter of the Cu5Fe3 adsorbent increased significantly to 2.428 nm, representing a remarkable enhancement compared with that of the Cu5 adsorbent.

**Figure 7:**
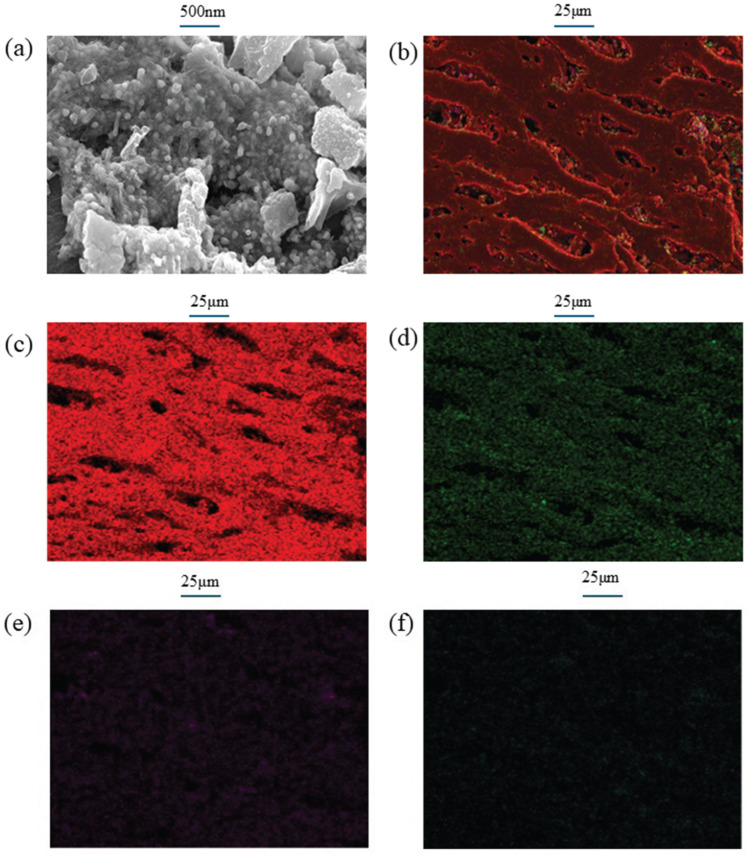
SEM, EDS, and mapping images of Cu5Fe3 adsorbent. (**a**) SEM image of Cu5Fe3 adsorbent (**b**) EDS image of Cu5Fe3 adsorbent, (**c**–**f**) mapping images of C, O, Cu, and Fe elements in Cu5Fe3 adsorbent. Among them, red represents carbon atoms, green represents oxygen atoms, pink represents copper atoms, and blue represents iron atoms.

To investigate the micromorphological characteristics and elemental distribution of the iron-loaded adsorbent, scanning electron microscopy (SEM) was employed to analyze the microstructure of the Cu5Fe3 sample, and energy-dispersive X-ray spectroscopy (EDS) was used for elemental mapping analysis. The results are shown in Figure 7. Figure 7a displays the SEM image of the Cu5Fe3 adsorbent. The sample exhibits an irregular granular morphology with a relatively uniform particle size distribution, indicating that the original framework structure of the material was not significantly damaged. Some fine particles are observed to agglomerate or adhere to the surfaces of larger particles [34,35]. This rough surface structure is beneficial for increasing the specific surface area, thereby providing more active sites for the adsorption process. Moreover, the stacking of particles creates a certain pore structure, which facilitates the diffusion of adsorbates within the material.

**Figure 8:**
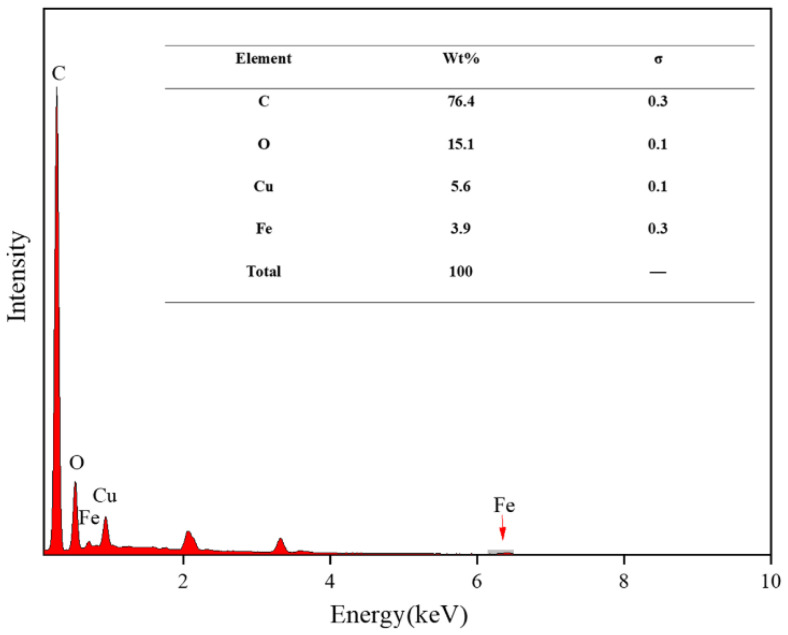
EDS spectrum of Cu5Fe3 adsorbent.

Figure 7b presents the electron image corresponding to the EDS mapping area. The EDS spectrum (as Figure 8) and the elemental mapping images of C, O, Cu, and Fe were obtained from this area. As shown in Figure 7c–f, C, O, Cu, and Fe are uniformly dispersed on the adsorbent, and the activated carbon material exhibits a distinct porous and layered stacking structure. This highly dispersed state indicates that the iron oxides and copper oxides form a composite structure with intimate contact on the material surface, rather than a simple physical mixture. Such a composite structure is conducive to interfacial charge transfer and the exertion of synergistic effects, which corroborates the Cu 2p binding energy shift observed by XPS.

**Figure 9:**
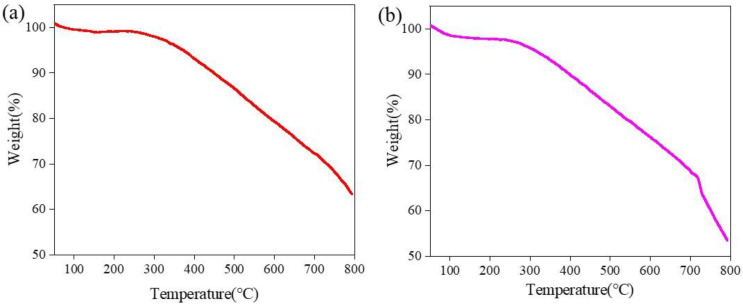
TGA curves of Cu5 and Cu5Fe3 adsorbents. (**a**) Cu5, (**b**) Cu5Fe3.

To evaluate the thermal stability of the Cu5Fe3 adsorbent, thermogravimetric analysis (TGA) was conducted under a nitrogen atmosphere. Figure 9 and Table 2 present the TGA curve and the residual mass percentage data for the Cu5Fe3 adsorbent. Based on the weight loss, the process can be divided into three stages.

In the range of 25–200 °C, the weight loss is mainly attributed to the removal of physically adsorbed water. Both samples exhibit low contents of physically adsorbed water, and the moisture absorption of the Cu5 adsorbent is slightly lower than that of Cu5Fe3.

In the range of 200–500 °C, the decomposition of surface functional groups and partial pyrolysis of the carbon skeleton predominantly occur. After 300 °C, the weight loss of both samples accelerates significantly, which is primarily ascribed to the decomposition of oxygen-containing functional groups (-OH, C=O) introduced by alkali modification at elevated temperatures. The more pronounced weight loss of Cu5Fe3 may be related to the stronger interaction between iron oxides and the carbon matrix [36,37].

In the range of 500–800 °C, further pyrolysis and condensation reactions of the carbon skeleton take place. The weight loss rates of both samples tend to level off in this stage, but the weight loss of Cu5Fe3 is notably higher. This indicates that at high temperatures, the copper–iron species catalyze carbon gasification or promote the reaction of carbon with residual oxygen or oxygen-containing functional groups to generate CO_2_ [38], thus exacerbating the pyrolytic loss of the carbon matrix. In contrast, the catalytic effect of copper species on carbon gasification is relatively weak, so Cu5 exhibits superior thermal stability. The above difference is mainly attributed to the higher chemical stability of copper oxides at high temperatures and the stronger catalytic effect of iron oxides on carbon gasification [39].

**Figure 10:**
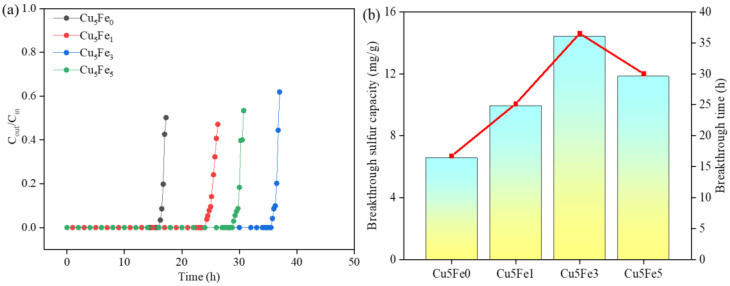
The adsorption effect of adsorbents with different iron loadings. (**a**) Breakthrough curve; (**b**) breakthrough time and breakthrough sulfur capacity. Among them, the line chart represents breakthrough sulfur capacity, and the bar chart represents breakthrough time.

A series of adsorbents with different Fe loadings were synthesized while keeping the adsorbent dosage and Cu loading constant. As shown in Figure 10, the adsorption performance exhibited a trend of first increasing and then decreasing. Among them, the Cu5Fe0 adsorbent displayed the lowest breakthrough time and breakthrough sulfur capacity. After introducing a small amount of iron, the breakthrough time and breakthrough sulfur capacity of the Cu5Fe1 adsorbent increased to 25.15 h and 9.941 mg/g, respectively. When the iron loading was further increased to Cu5Fe3, the adsorption performance reached the optimum, with a breakthrough time of 36.5 h and a breakthrough sulfur capacity of 14.424 mg/g. Further increasing the iron loading to Cu5Fe5 caused the breakthrough time and breakthrough sulfur capacity to decrease to 30 h and 11.833 mg/g, respectively. These results indicate that loading an appropriate amount of iron can enhance the adsorption performance, whereas excessive iron loading leads to the agglomeration of iron oxides on the adsorbent surface, reduces the number of effective active sites, and obstructs the exposure of copper species, thereby resulting in decreased adsorption performance [40].

### 3.3. Effect of Different Bimetallic Loading Ratios on Adsorbent Performance

**Figure 11:**
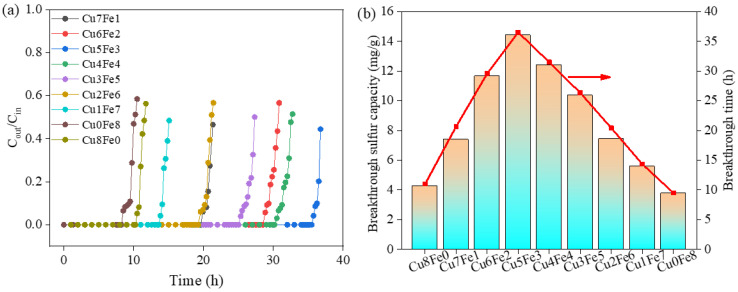
Adsorption effect of adsorbents prepared with different bimetallic loading ratios. (**a**) Breakthrough curve; (**b**) breakthrough time and breakthrough sulfur capacity. Among them, the bar chart represents the breakthrough sulfur capacity, and the line chart represents the breakthrough time.

In the previous section, the effect of iron loading on the adsorption performance was investigated while keeping the copper loading constant. In this section, with the total Cu-Fe loading fixed at 8% by mass, the optimal Cu/Fe bimetallic ratio was explored. A series of adsorbents with different Cu/Fe ratios were prepared, and dynamic adsorption experiments were carried out using the same adsorbent dosage. The results are shown in Figure 11.

Figure 11a presents the breakthrough curves of nine adsorbents with different Cu/Fe ratios. It can be observed that the monometallic adsorbents Cu8Fe0 and Cu0Fe8 both reached the breakthrough concentration rapidly. Among them, the breakthrough curve of Cu0Fe8 rose earliest, exhibiting the shortest breakthrough time and a steep profile. This indicates that under high space velocity, the adsorbent possesses limited active sites, resulting in weak adsorption capacity for the target adsorbate and low utilization efficiency [33,41].

In the copper-rich region (Cu7Fe1 and Cu6Fe2 adsorbents), the introduction of a small amount of iron caused a noticeable rightward shift of the breakthrough curves compared with Cu8Fe0 and prolonged the breakthrough times, indicating that the addition of an appropriate amount of iron increased the number of effective active sites and that a synergistic effect between copper and iron began to emerge. In the intermediate ratio region (Cu5Fe3, Cu4Fe4, and Cu3Fe5 adsorbents), the breakthrough curves shifted further to the right, with Cu5Fe3 exhibiting the best adsorption performance and the longest breakthrough curve. This demonstrates that, under a fixed total metal loading, the synergistic effect is fully exerted when the Cu/Fe mass ratio is close to 5:3 or 1:1. Cu4Fe4 also showed good adsorption performance, though slightly inferior to Cu5Fe3.

In the iron-rich region (Cu2Fe6 and Cu1Fe7), as the iron proportion continued to increase, the breakthrough curves shifted leftward and the breakthrough times gradually shortened. This indicates that excessive substitution of copper by iron alters the nature of the active sites, weakens the dominant role of copper, and diminishes the synergistic effect, leading to a decline in adsorption performance. For the Cu0Fe8 adsorbent, its breakthrough curve was similar to that of the pure copper adsorbent (Cu8Fe0), again appearing on the left side with the shortest breakthrough time. This suggests that the adsorption capacity of the monometallic iron-based adsorbent is also limited and is far inferior to that of the optimal copper–iron composite adsorbent.

Figure 11b presents the breakthrough times and breakthrough sulfur capacities of the nine adsorbents with different Cu/Fe mass ratios. As can be seen, the breakthrough sulfur capacity exhibits a trend of first increasing and then decreasing with the increase in the iron loading ratio (or the decrease in the copper loading ratio). Among all samples, Cu5Fe3 shows the best adsorption performance, with a breakthrough time of 36.5 h and a breakthrough sulfur capacity of 14.424 mg/g. The above results indicate that under the current adsorption conditions, the optimal Cu/Fe mass ratio is 5:3, at which the synergistic effect between the two metals reaches its maximum.

### 3.4. Effect of Operating Conditions on Adsorption Performance

**Figure 12:**
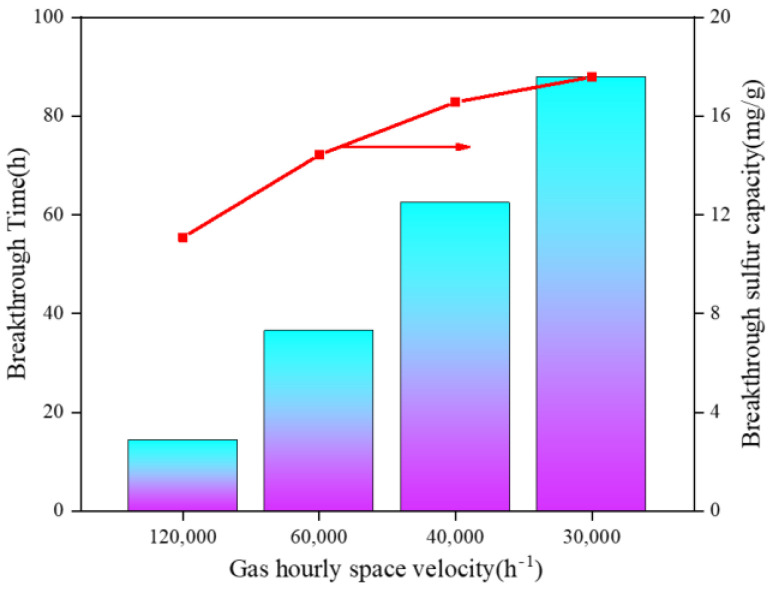
The effect of different air velocities on the adsorption efficiency of SO_2_. Among them, the bar chart represents the breakthrough time, and the line chart represents the breakthrough sulfur capacity. Adsorption conditions: 0.04 g adsorbent, 25 °C, inlet concentration 1 ppm, outlet concentration 0.1 ppm, space velocity 120,000–30,000 h^−1^, inlet flow rate 100 mL/min.

Figure 12 presents the adsorption performance of the adsorbent at different volumetric space velocities. The results indicate that as the space velocity decreases, both the breakthrough time and the breakthrough sulfur capacity gradually increase [42]. The optimal adsorption performance is achieved at a volumetric space velocity of 30,000 h^−1^, with a breakthrough time of 87.917 h and a breakthrough sulfur capacity of 17.587 mg/g. At a space velocity of 120,000 h^−1^, the residence time of the gas in the bed is too short, and SO_2_ molecules cannot sufficiently diffuse into the internal pore channels of the adsorbent to contact the active sites, leading to a decline in adsorption efficiency. When the space velocity is reduced to 30,000 h^−1^, the external diffusion resistance is significantly diminished, enabling SO_2_ molecules to effectively pass through the boundary layer and reach the external surface of the adsorbent. At the same time, a sufficient residence time is ensured, which is favorable for the diffusion of SO_2_ molecules into the micropores within the particles [43].

**Figure 13:**
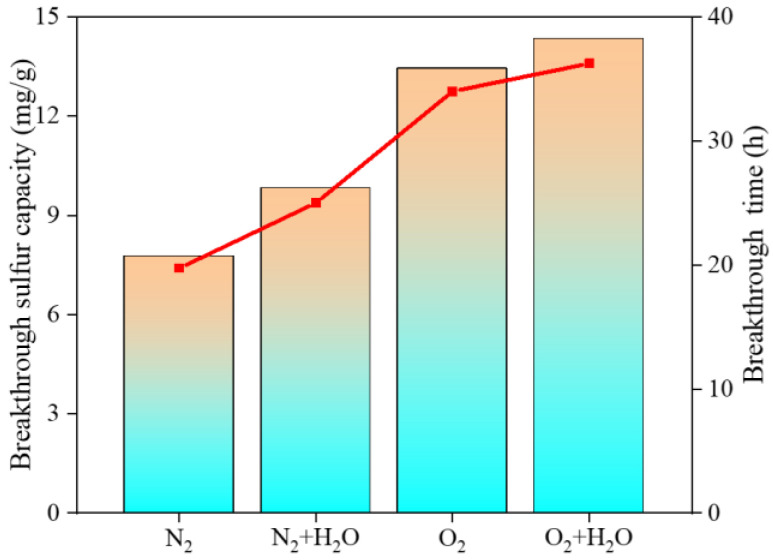
The effect of adsorbents on the adsorption performance of SO_2_ under different atmospheric conditions. Among them, the line chart represents the breakthrough of sulfur capacity, and the bar chart represents the breakthrough time. Adsorption conditions: 0.04 g adsorbent, 25 °C, inlet concentration of 1 ppm, outlet concentration of 0.1 ppm, space velocity of 60,000 h^−1^, inlet flow rate of 100 mL/min. Among them, the volume fraction of O_2_ is 20%, and the relative humidity is 70%.

Figure 13 illustrates the effect of different atmospheres on the SO_2_ adsorption performance of the adsorbent. As can be seen, under a pure N_2_ atmosphere, both the breakthrough time and the breakthrough sulfur capacity are relatively low. Under this condition, the adsorption primarily relies on the capture of SO_2_ by metal oxides to form sulfites; however, owing to the high space velocity, the SO_2_ capture capacity is limited and highly efficient adsorption is difficult to achieve [44]. When water vapor is introduced into the system, the adsorption performance is slightly enhanced compared with that under the pure N_2_ atmosphere, which can be attributed to the dissociative adsorption of water molecules on the metal oxide surface to form hydroxyl groups. These hydroxyl groups can serve as favorable active sites and react with the acidic gas SO_2_ [25,45]. In an O_2_ atmosphere, both the breakthrough time and the breakthrough sulfur capacity are significantly improved, exhibiting an increasing trend. This is mainly because O_2_ facilitates the further oxidation of the adsorbed sulfite to sulfate, which is then immobilized on the adsorbent surface [46]. Under the coexisting O_2_ and H_2_O atmosphere, the breakthrough time and breakthrough sulfur capacity increase further, achieving the best adsorption performance with a breakthrough time of 36.5 h and a breakthrough sulfur capacity of 14.424 mg/g. The above results demonstrate that the coexistence of O_2_ and H_2_O significantly promotes the adsorption process [47].

### 3.5. Cyclic Stability and Deactivation Analysis

**Figure 14:**
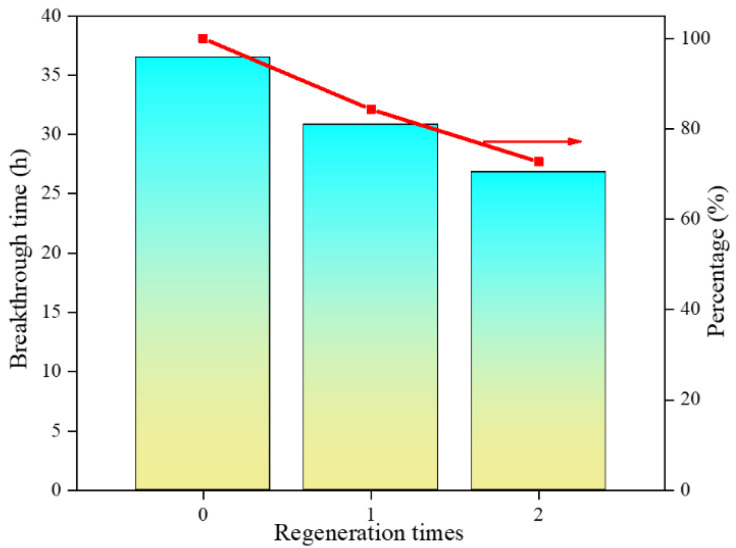
Cycling test of Cu5Fe3 adsorbent. Among them, the bar chart represents the breakthrough time, and the line chart represents the cyclic regeneration activity of the adsorbent Adsorption conditions: 0.04 g adsorbent, 25 °C, inlet concentration of 1 ppm, outlet concentration of 0.1 ppm, space velocity of 60,000 h^−1^, inlet flow rate of 100 mL/min, coexisting atmosphere of O_2_ and H_2_O.

In industrial production, the stability and reusability of the adsorbent are key factors affecting the operating cost. Based on the discussion in the previous sections regarding the synthesis conditions and the optimal loading ratio, the Cu5Fe3 adsorbent was selected for further investigation. Considering that excessively low space velocity would significantly prolong the breakthrough time and increase the time cost, cyclic regeneration experiments were carried out at a volumetric space velocity of 60,000 h^−1^ to evaluate its cyclic regeneration performance. Figure 14 presents the cyclic regeneration performance of the Cu5Fe3 adsorbent. It can be observed that the overall adsorption performance exhibits a declining trend during the regeneration process. After two cyclic tests, the breakthrough time and breakthrough sulfur capacity decreased to 26.833 h and 10.497 mg/g, respectively, corresponding to an adsorption activity of 72.7% of that of the fresh adsorbent. This result may be attributed to the irreversible deactivation of some active sites after regeneration, leading to a decrease in adsorption capacity. Meanwhile, with increasing regeneration cycles, the regeneration process may fail to completely remove the continuously accumulated sulfates on the adsorbent surface, causing the active sites to be covered or blocked and resulting in a continuous decline in adsorption performance [48].

**Figure 15:**
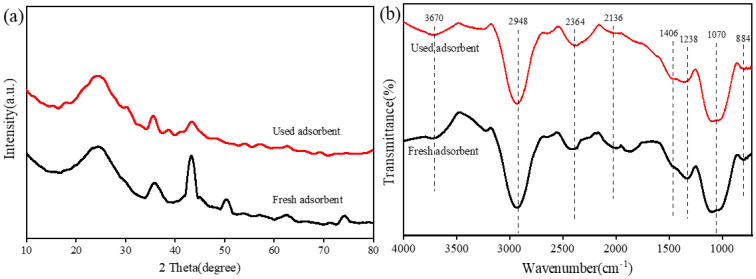
XRD and FT-IR spectra of Cu5Fe3 adsorbent before and after reaction. (**a**) XRD spectra of fresh adsorbent and used adsorbent, (**b**) FT-IR spectra of fresh adsorbent and used adsorbent.

Figure 15a shows the XRD patterns of the Cu5Fe3 adsorbent before and after the desulfurization reaction. It can be observed that the XRD pattern of the used adsorbent changed significantly. Compared with the fresh sample, the characteristic peaks of CuO and Fe_2_O_3_ weakened substantially and nearly disappeared, indicating a decrease in the crystallinity of the active components or a phase transformation. Meanwhile, the background in the low-angle region (2θ < 30°) increased noticeably, and weak new diffraction peaks appeared near 25.1°, 29.8°, and 38.2°. By comparison with standard reference cards, these new peaks can be identified as characteristic diffraction peaks of CuSO_4_ and Fe_2_(SO_4_)_3_. The above results indicate that sulfate species were formed during the reaction, and the newly formed sulfates exist mainly in an amorphous or highly dispersed nanoparticle form.

Figure 15b presents the Fourier transform infrared spectra of the Cu5Fe3 adsorbent before and after the reaction. Both sets of samples exhibit distinct absorption bands at 3670 cm^−1^, 2948 cm^−1^, 2364 cm^−1^, 2136 cm^−1^, 1394 cm^−1^, 1238 cm^−1^, 1070 cm^−1^, and 884 cm^−1^. The positions, shapes, and relative intensities of the absorption bands in the two samples are largely consistent, indicating that the original skeletal structure of the activated carbon material remains unchanged after the desulfurization reaction. Combined with X-ray diffraction and infrared spectroscopy results, it can be inferred that during the desulfurization process of the Cu5Fe3 adsorbent, the oxidized copper and iron in the active components react with SO_2_ to form amorphous sulfates, while the skeletal structure of the activated carbon support remains stable.

**Figure 16:**
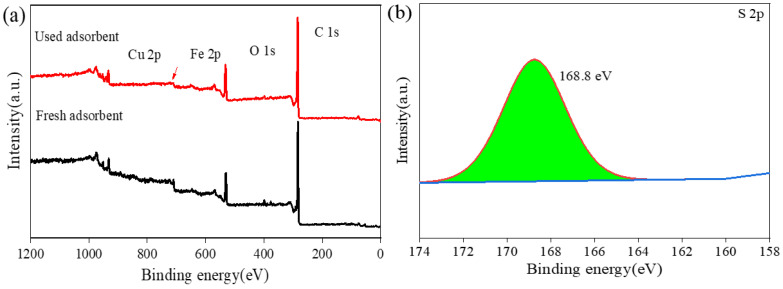
XPS spectra of Cu5Fe3 adsorbent before and after reaction. (**a**) XPS spectra of fresh adsorbent and adsorbent after reaction. (**b**) The S 2p fine spectrum of the adsorbent after the reaction.

XPS analysis was employed to investigate the changes in chemical composition and chemical states of the used adsorbent, as shown in Figure 16. Figure 16a displays the survey spectra of the adsorbent before and after the reaction. The characteristic peaks of Cu 2p, Fe 2p, O 1s, and C 1s are detected in both spectra, indicating that the major constituent elements of the adsorbent remained stable after the reaction. A comparison of the O 1s peaks reveals a slight increase in the O 1s intensity after adsorption. In addition, a new weak signal peak appears in the spectrum of the used adsorbent at a binding energy of approximately 168.8 eV, which is assigned to the S 2p signal, indicating the deposition of sulfur-containing species on the adsorbent surface during the reaction. However, owing to its low signal intensity, it is not prominent in the XPS survey spectrum.

Furthermore, Figure 16b shows the high-resolution S 2p spectrum of the used adsorbent, which exhibits a distinct single peak at 168.8 eV. According to the XPS standard database, this binding energy corresponds to the S 2p_3/2_ characteristic peak of sulfate (SO_4_^2−^) or sulfite (SO_3_^2−^). Combined with the presence of O_2_ under the reaction conditions, it can be inferred that gaseous SO_2_ was captured by the adsorbent and subsequently oxidized to high-valence sulfate species on the surface, thereby achieving fixation and removal on the adsorbent.

## 4. Conclusions

(1) The introduction of Fe into the Cu/AC-OH adsorbent significantly enhances the adsorption performance for ultra-low-concentration SO_2_. The introduced Fe forms Fe-O-Cu structures with Cu, strengthening the electronic interactions and increasing oxygen vacancies, thereby improving the dispersion of the active components and the number of adsorption sites. The Cu5Fe3 adsorbent with a Cu loading of 5% by mass and an Fe loading of 3% by mass exhibits the optimal adsorption activity, achieving a breakthrough time of 36.5 h and a breakthrough sulfur capacity of 14.424 mg/g.

(2) Decreasing the space velocity prolongs the gas residence time, which facilitates diffusion and adsorption. The coexistence of O_2_ and H_2_O exerts a synergistic promoting effect on SO_2_ adsorption, representing an important condition for achieving efficient desulfurization.

(3) After two regeneration cycles, the activity of the Cu5Fe3 adsorbent decreases to 72.7%. The deactivation is mainly attributed to the accumulation of sulfates covering the active sites and the agglomeration or loss of the active components.

(4) Characterizations including XRD, XPS, FT-IR, and SEM-EDS confirm the successful loading and uniform distribution of the metals, the formation of sulfates after the reaction, and the stability of the carbon framework structure.

(5) The Cu-Fe bimetallic-modified coconut shell activated carbon demonstrates considerable application potential for the deep purification of ultra-low-concentration SO_2_. Further improvements in the regeneration strategy and anti-deactivation performance will be the key focus of future research.

## Figures and Tables

**Table 1:** Comparison of different structural parameters of Cu5 and Cu5Fe3 adsorbents.

Adsorbent	S_BET_ (m^2^·g^−1^)	Pore Volume (cm^3^·g^−1^)	Average Pore Size (nm)
Cu5	629.970	0.311	2.100
Cu5Fe3	613.297	0.255	2.428

**Table 2:** Percentage of remaining mass of Cu5 and Cu5Fe3 adsorbents at different temperatures.

Adsorbent	200 °C	300 °C	400 °C	500 °C	600 °C	700 °C	800 °C
Cu5	99.18%	98.01%	93.10%	86.65%	79.40%	72.35%	63.48%
Cu5Fe3	97.76%	95.84%	89.92%	82.96%	76.15%	68.85%	53.38%

## Data Availability

The original contributions presented in this study are included in the article. Further inquiries can be directed to the corresponding author.
